# From Species Identification to Empirical Therapy: A Machine Learning and Rule-Based Decision Support Framework for Antifungal Resistance Prediction in ICU *Candida* Infections

**DOI:** 10.3390/medsci14020319

**Published:** 2026-06-15

**Authors:** Madalina (Preda) Solomon, Beatrice Mahler, Lia-Mara Ditu, Oana Popescu, Corina-Aurelia Zugravu, Loredana Sabina Cornelia Manolescu

**Affiliations:** 1 Department of Microbiology, Parasitology and Virology, Faculty of Nursing, Carol Davila University of Medicine and Pharmacy, 020021 Bucharest, Romania; madalina.preda@umfcd.ro (M.S.); loredana.manolescu@umfcd.ro (L.S.C.M.); 2 Clinical Laboratory of Medical Microbiology, Marius Nasta Institute of Pneumology, 050159 Bucharest, Romania; 3 Cardiothoracic Department, Faculty of Medicine, Carol Davila University of Medicine and Pharmacy, 020956 Bucharest, Romania; beatrice.mahler@umfcd.ro; 4 Pneumology Department, Marius Nasta Institute of Pneumology, 050159 Bucharest, Romania; 5 Department of Botany and Microbiology, Faculty of Biology, University of Bucharest, 060101 Bucharest, Romania; lia-mara.ditu@bio.unibuc.ro; 6 MICROGEN Research Centre, Faculty of Biology, University of Bucharest, 060101 Bucharest, Romania; 7 National Reference Laboratory of Tuberculosis, Marius Nasta Institute of Pneumology, 050159 Bucharest, Romania; oana.popescu@marius-nasta.ro; 8 Department of Hygiene and Nutrition, Carol Davila University of Medicine and Pharmacy, 050463 Bucharest, Romania; 9 National Institute of Public Health, 050463 Bucharest, Romania

**Keywords:** antifungal resistance, intensive care unit, machine learning, *Candida*

## Abstract

**Objectives**: When a *Candida* species is identified in an ICU patient, susceptibility results are typically available in 24–72 h. In this study, we built a machine learning model using four variables available at identification to estimate resistance probability in real time. **Methods**: We analysed 747 fungal isolates from 725 ICU patients (January 2021–March 2026). We trained and compared a Random Forest and a Logistic Regression model, evaluating both with temporal cross-validation, permutation feature importance, three-category (S/I/R) prediction, and calibration analysis. **Results**: Multidrug resistance doubled from 24.5% (2021) to 51.1% (2025), and *Candida auris* grew eight-fold in three years. Random Forest reached AUC 0.885 on the held-out test set and 0.848 on prospective 2024–2025 data (Brier score 0.093). Species identity and drug choice together explained 87% of predictive signal. Local *C. albicans* fluconazole resistance (~16%) far exceeded the ECMM European figure of 0%, and *C. krusei* was four times more prevalent than the continental average. **Conclusions**: A four-variable model may provide calibrated resistance estimates during the critical gap before susceptibility results return, though performance reflects predominantly deterministic species–drug patterns rather than complex learned biology. Overall performance was comparable to a rule-based lookup table, confirming that the majority of predictive signal derives from established species–drug susceptibility patterns. Meaningful added value is limited to temporal trend tracking and improved prediction where resistance is acquired rather than intrinsic (*C. albicans*, *C. tropicalis* hard-subset AUC 0.929 vs. rule-based 0.899). The model complements a local antifungal testing; it does not replace one.

## 1. Introduction

*Candida* spp. are common commensal organisms in the skin and gut microbiota [1]. In some cases, it can cause pathologies with a wide range of manifestations from asymptomatic to severe, invasive candidiasis [1,2]. Invasive candidiasis has a high mortality, approximately 40–50% [2]. Invasive candidiasis can represent a problem in healthcare associated infections as well, in some multicentric studies being reported as 9.5% of the cases are caused by *Candida* spp. [3,4].

Patients with these severe infections can be treated with antifungal medications from a few different pharmacological classes [5,6]. Since *Candida* does not share resistance mechanisms, acquired resistance either develops in response to an antifungal selection pressure in a single patient or, less frequently, results from the horizontal transmission of resistant strains across patients [5]. *Candida* species’ growing resistance to azoles and echinocandins is a major issue in clinical settings across the globe [7,8,9]. Multidrug resistance to azoles, echinocandins, and polyenes is rare, but it has been reported in a number of Candida species, most notably *Candida glabrata* and, more recently, *Candida auris* [5,10]. The World Health Organization has listed *C. auris* as a critical priority pathogen on its most recent list of fungi [11]. With fatality rates ranging from 30% to 72%, *C. auris* infections have been documented in the bloodstream and, less frequently, in the abdomen and cerebrospinal fluid [12]. It might be difficult to diagnose *C. auris* infections, especially when using traditional methods [10,12].

Most of the cases in intensive care units, antifungal therapy is required and each delayed hour may cost lives, especially considering the time required to obtain antifungal testing results after species identification [8,13]. To avoid delays, alternative diagnostic tests are required.

Application of machine learning (ML) has increased in the medical field, including in approaching the subject of antimicrobial resistance [14,15]. However, most of the studies are covering the bacterial resistance to antibiotics [16], and in the mycology domain, studies are including genomic data, not routinely and rapid available data in most of the settings [17].

A key conceptual distinction underlies this work. Some species–drug combinations carry intrinsic resistance designations per EUCAST—meaning resistance is fixed, species-level, and does not require susceptibility testing to predict (*C. krusei*–fluconazole, *C. glabrata*–fluconazole). For these combinations, any rule-based lookup table already achieves near-perfect prediction. By contrast, acquired resistance—particularly in *C. albicans* and *C. tropicalis*—is not predictable from species identity alone and represents the clinically uncertain territory where probabilistic modelling adds genuine value. Published machine learning approaches in antifungal resistance have largely relied on whole-genome sequencing data [17], which is unavailable at the time of clinical decision-making in most settings. Tools based solely on routine microbiological variables—species, drug, isolation year, and sample type—have not been prospectively validated. Within antimicrobial stewardship, local antibiograms and species-drug lookup tables represent the current standard; the present model is designed to complement rather than replace these tools, by adding temporal calibration and calibrated probability estimates for acquired resistance scenarios where static tables offer no guidance.

Until now, to the best of our knowledge, there is no ML model prospectively validated, applicable at the time of identifying the species. Local stewardship tools are recommended by the major and recent guidelines [18]. The present study aimed to (1) characterise the antifungal resistance landscape in *Candida* spp. isolates from a tertiary-care ICU in Eastern Europe over a five-year period, benchmarking findings against European surveillance data; (2) develop and validate a machine learning model capable of generating real-time, probabilistically calibrated non-susceptibility estimates at the moment of fungal species identification, using only four variables available in routine microbiological practice, without the need for genomic sequencing, specialised mass spectrometry, or individual patient clinical data, and (3) to quantify the incremental value of machine learning over a rule-based species-drug lookup table, identifying the clinical scenarios where probabilistic modelling offers guidance beyond established intrinsic resistance patterns.

## 2. Materials and Methods

### 2.1. Study Design, Setting and Data Source

A retrospective, observational study was conducted at a tertiary-care teaching hospital in Bucharest, Romania. The institution is a national referral centre with a mixed medical–surgical ICU serving patients with various respiratory disorders—the population in which invasive candidiasis carries the highest mortality.

Data were extracted from the laboratory information system for the period January 2021 through March 2026. Each record included: a de-identified patient code, the calendar year of isolation, the biological sample type as free-text, the fungal species as reported by the microbiology laboratory (VITEK-2 biochemical identification, supplemented by MALDI-TOF where available), and MIC values with original automated interpretations for up to six antifungal agents: fluconazole, voriconazole, caspofungin, micafungin, amphotericin B, and flucytosine. After data cleaning, 747 isolate records from 725 unique patients were retained. The 22 patients with more than one isolate record contributed sequential or simultaneous episodes and were retained as separate observations.

Throughout this manuscript, fungal species are referred to using the traditional *Candida* nomenclature for clinical clarity, acknowledging that several species have been reclassified under updated taxonomic designations, *C. glabrata* (*Nakaseomyces glabrata*), *C. krusei* (*Pichia kudriavzevii*), *C. kefyr* (*Kluyveromyces marxianus*), and *C. lusitaniae* (*Clavispora lusitaniae*), and *C. dubliniensis* retains its name. The traditional nomenclature is retained throughout as it remains the standard in clinical microbiology reporting and in the EUCAST breakpoint tables used for susceptibility interpretation in this study.

### 2.2. Antifungal Susceptibility Re-Interpretation

All MIC values were re-categorised using the EUCAST Antifungal Clinical Breakpoint Table v12.1 (valid from 10 April 2026) [19]. Throughout this manuscript, the following terminology is used consistently: non-susceptibility refers to the combined I + R category per EUCAST v12.1 and constitutes the binary prediction target; confirmed acquired resistance refers exclusively to the R category (MIC above the EUCAST R breakpoint); intrinsic resistance refers to fixed species-level EUCAST designations independent of MIC testing. The term “resistance” alone is used only in a general clinical context and not as a substitute for either of the above. Categories assigned were: S (Susceptible, standard dosing regimen), I (Susceptible, Increased Exposure—not a marker of acquired resistance), and R (Resistant). CLSI M27-Ed5 (2022) [20] breakpoints were applied for species–drug combinations not covered by EUCAST, designated with a CLSI suffix. Caspofungin susceptibility was inferred from micafungin per EUCAST Note 2. Records with missing or indeterminate MIC values were classified as ND (not determined) and excluded from susceptibility modelling.

One interpretive limitation requires explicit acknowledgement: EUCAST v12.1 sets the S breakpoint for micafungin at MIC ≤ 0.03 mg/L and for voriconazole at ≤0.06 mg/L, whereas the automated susceptibility system reports MICs only down to ≤0.06 mg/L (micafungin) and ≤0.12 mg/L (voriconazole). Isolates whose true MIC falls below the resistance threshold are nonetheless assigned to category I by strict EUCAST v12.1 application, not because of acquired resistance, but because the instrument cannot resolve sub-breakpoint values. This limitation is acknowledged throughout the results where relevant.

### 2.3. Epidemiological and Statistical Analyses

Descriptive statistics were computed for species distribution, sample type, and antifungal testing coverage. Temporal trends were examined year-by-year from 2021 to 2025 (2026 excluded, *n* = 18, collection ongoing). MDR was defined as non-susceptibility to two or more antifungal drug classes tested simultaneously. MIC distributions were assessed for bimodality, the microbiological signature of a mixed wild-type plus non-wild-type population. Co-resistance pair frequencies were computed as the number of isolates simultaneously classified non-susceptible to each pairwise drug combination. Full epidemiological analyses are presented in Supplementary Figures S1–S4. All analyses were performed in Python 3.12.

### 2.4. Machine Learning—Model Development and Evaluation

The prediction problem was framed as binary classification: non-susceptibility (I or R per EUCAST v12.1, coded as 1) versus susceptibility (S, coded as 0). Observations with ND or IE (insufficient evidence) interpretations were excluded, yielding 1873 isolate-drug observations from 583 unique patients, of which 31.8% were non-susceptible (I + R). Records with missing, indeterminate (ND), or insufficient-evidence (IE) interpretations were excluded from modelling entirely; no imputation was performed.

Data were restructured from wide format (one row per isolate, multiple antifungal columns) to long format (one row per isolate–antifungal pair), yielding a maximum of six observations per isolate depending on testing coverage.

Four predictors were used: (1) *Candida* species, label-encoded; (2) antifungal drug tested, label-encoded; (3) isolation year, treated as a continuous numeric variable; (4) sample type, ordinal-encoded into five categories (respiratory, urine, blood culture, sterile fluid, other). All four variables are available immediately at species identification, before susceptibility testing begins.

To avoid pseudo-replication and patient-level data leakage—the same patient’s isolates contributing observations to both training and test sets across multiple antifungal drugs—splitting was performed at the patient level using GroupShuffleSplit (scikit-learn), ensuring no patient appears in both training and test partitions. This yielded training (*n* = 1408 observations, 437 patients) and test (*n* = 465 observations, 146 patients) sets. Patient overlap between partitions was verified to be zero. The 22 patients who contributed more than one isolate record represented sequential or simultaneous episodes; their repeated records were treated as independent observations within the same patient group, ensuring all records from a given patient remained in the same partition.

Two classifiers were selected as representative of the interpretable-model spectrum: Random Forest (RF; ensemble of decision trees, non-linear, resistant to correlated features) and Logistic Regression (LR; linear, more regularised, typically more robust under distributional shift). Both were trained with 200 trees/maximum depth 8 (RF) and L2 regularisation (LR), with balanced class weights to compensate for the 31.8% minority class. Random Forest and Logistic Regression were specifically chosen over gradient boosting approaches (XGboost V.2.0, LightGBM V.4.6.0) because they offer superior interpretability—permutation importance and coefficient inspection—which is essential for clinician trust in a clinical decision support context. Performance was assessed using AUC (primary metric, with 1000-iteration bootstrap 95% confidence intervals), accuracy, precision, recall, F1-score, and 5-fold Group K-Fold cross-validation at the patient level. All analyses were implemented in scikit-learn 1.x (Python 3.12, random_state = 42).

A rule-based comparator model was constructed to evaluate whether the machine learning classifiers add discriminative value beyond simply encoding known intrinsic species-drug susceptibility patterns. The rule-based model predicted the empirical non-susceptibility rate for each species–drug pair observed in the training set, without incorporating year or sample type. This serves as a benchmark representing the performance achievable by a clinician with access to a local antifungigram table but without the temporal and contextual modelling provided by machine learning.

### 2.5. Advanced and Sensitivity Analyses

Six complementary analyses extended the following standard evaluation. (1) Permutation feature importance: Each feature independently shuffled 30 times on the patient-level test set, with mean AUC decrease recorded as the importance metric. (2) Temporal cross-validation: trained on 2021–2023 (*n* = 1043), tested on 2024–2025 (*n* = 799), simulating prospective deployment, referred to as pseudo-prospective given that both periods derive from the same institution. (3) Three-category prediction (S/I/R): A separate Random Forest trained to distinguish all three EUCAST categories. (4) Calibration: Reliability was assessed by plotting calibration curves using 8-bin isotonic regression on the test set, comparing mean predicted probability against observed non-susceptibility rate per bin. Brier score was computed for both classifiers and compared against a naïve baseline that predicts the marginal prevalence for all observations. Post hoc calibration using Platt scaling is recommended for deployment but was not applied in the primary analysis. (5) Sensitivity analysis excluding instrument-truncated combinations: *C. albicans* voriconazole and micafungin observations were excluded to assess model performance independently of the VITEK-2 lower detection limit artefact. (6) Hard-subset analysis: Model evaluated exclusively on *C. albicans* and *C. tropicalis*, the two species without intrinsic resistance designations for the tested drugs, to estimate performance on genuinely uncertain clinical predictions. Bootstrap 95% confidence intervals (1000 iterations) were computed for all primary AUC estimates.

### 2.6. European Benchmarking

Local resistance rates were compared against the ECMM *Candida* III Study [18]: 399 candidemia isolates from 41 centres in 17 European countries, 2018–2022, with MIC determination by EUCAST broth microdilution in central reference laboratories. Comparisons are descriptive; formal statistical testing was not performed given the methodological heterogeneity between datasets (sample types, MIC methodology, breakpoint versions, and study periods).

### 2.7. Ethics and Data Access

The study protocol was approved by the Ethics Committee of the Marius Nasta Institute of Pneumology, Bucharest, Romania (approval no. 23343/17 October 2023), and data access was authorised under approval no. 23113/12 October 2023. The study was conducted in full compliance with the Declaration of Helsinki and applicable national regulations governing the use of retrospective clinical data. Patient records were de-identified prior to extraction from the laboratory information system; no personal identifiers were included in or recoverable from the analytical dataset. Informed consent was waived by the Ethics Committee of the Marius Nasta Institute of Pneumology, given the retrospective design of the study, the use of fully anonymized data, and the absence of any patient intervention or tissue collection, in accordance with applicable institutional and national guidelines.

## 3. Results

### 3.1. Epidemiological Profile and Resistance Landscape

**Figure 1:**
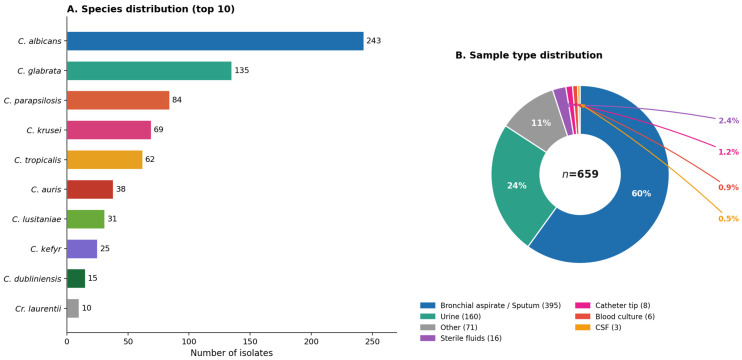
Species distribution and sample types. (**A**) Distribution of 747 ICU isolates by *Candida* species (top 10 shown), 2021–2026. (**B**) Proportions by biological sample type. Bronchial aspirate/sputum accounted for more than half of all specimens. Twenty-two distinct fungal species were identified.

Over the five-year study period, 747 fungal isolates were collected from 725 unique ICU patients, representing 22 distinct fungal species. The multidrug resistance rate doubled from 24.5% in 2021 to 51.1% in 2025, and *C. auris* increased eight-fold over three years—from zero isolates in 2021 to 16 in 2025. Full epidemiological analyses, including MDR trends, MIC distributions, and co-resistance patterns, are presented in Supplementary Figures S1–S4. The findings most directly relevant to the machine learning model are summarised here. Figure 1 shows the species distribution across the 747 ICU isolates and the breakdown by sample type.

*C. albicans* remained the most common species (32.5%), though at the lower end of what European surveillance typically reports. More clinically significant is the combined 27.3% share held by *C. glabrata* and *C. krusei*—two species with inherently reduced azole susceptibility, meaning that more than one in four isolates carries a built-in limitation on empirical azole therapy before a single susceptibility test has been run. *C. auris* accounted for just 5.1% of isolates overall, but that figure masks an alarming trajectory detailed in Supplementary Figure S1. Thirteen patients yielded isolates of two or more distinct fungal species simultaneously, including three with exceptional co-infection profiles described in the Supplementary Material.

**Figure 2:**
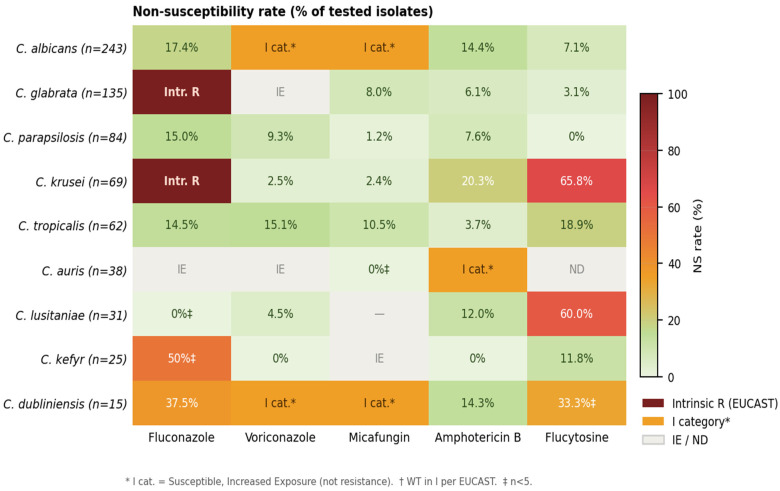
Resistance heatmap—Species × drug, EUCAST v12.1. Non-susceptibility rates (%) per species × antifungal combination, re-interpreted using EUCAST v12.1. Dark burgundy = intrinsic resistance; orange = I category (Susceptible, Increased Exposure—not acquired resistance); white-grey = insufficient evidence or not tested. Rates based on *n* < 5 isolates (‡) should be interpreted with caution.

These species proportions define the baseline resistance probability that the machine learning model must learn to navigate; Figure 2 maps that resistance at the drug level.

*C. krusei* fluconazole carries an intrinsic resistance designation—azoles must never be used regardless of the MIC, and the model assigns near-100% non-susceptibility probability to this combination. *C. glabrata* fluconazole is effectively ruled out by the same logic: the EUCAST v12.1 breakpoint (S ≤ 0.001 mg/L) places the entire wild-type population in category I. The orange cells labelled I cat. for *C. albicans* voriconazole and micafungin are not a resistance signal; they reflect a technical limitation of the automated system, whose lower detection limit prevents it from resolving MIC values below the EUCAST v12.1 susceptibility threshold. The truly concerning cells are those in red: *C. krusei* flucytosine at 65.8%, *C. tropicalis* across multiple drug classes (14.5–18.9%), and scattered amphotericin B resistance discussed further in Supplementary Figure S2.

Together, Figure 1 and Figure 2 define what the model must learn. The next section reports how well it succeeds.

### 3.2. Machine Learning Model Performance

**Figure 3:**
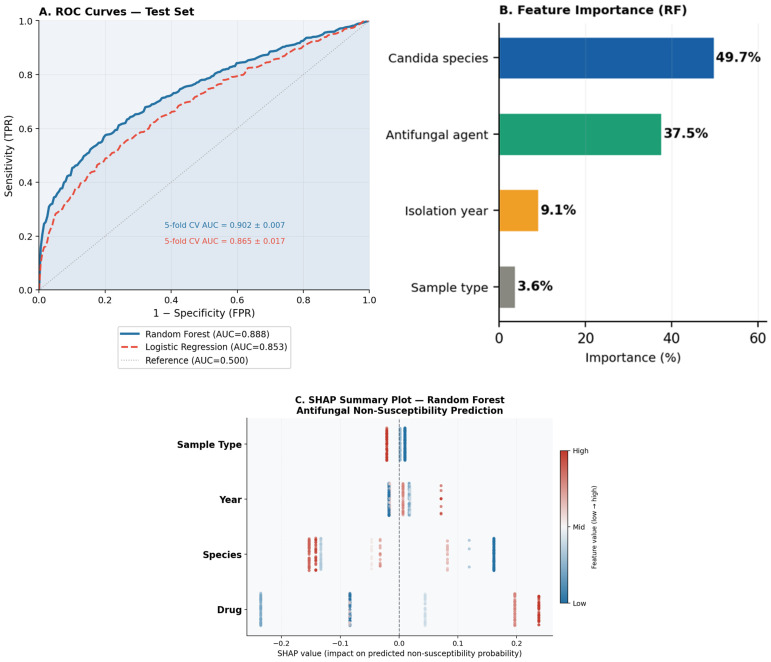
ROC curves and feature importance. (**A**) Receiver operating characteristic (ROC) curves for the Random Forest (solid blue, AUC = 0.893, 95% CI 0.852–0.930) and Logistic Regression (dashed orange, AUC = 0.852, 95% CI 0.801–0.895) models on the patient-level held-out test set (*n* = 465 observations, 146 patients, zero patient overlap with training set). The rule-based comparator model (species–drug empirical rates from training data) achieved AUC = 0.896 (95% CI 0.854–0.936), shown as the dotted grey line. 5-fold Group K-Fold patient-level cross-validation AUC: 0.902 ± 0.014. CV = Cross-Validation (**B**) Variable importance scores from the Random Forest model (mean decrease in node impurity across all 200 trees). (**C**) SHAP summary plot for the Random Forest model. Each point represents one isolate–drug observation from the held-out test set (*n* = 300 sampled observations). The x-axis shows the SHAP value—the contribution of each feature to the predicted non-susceptibility probability relative to the baseline expectation (0.298). Points to the right of zero increase predicted non-susceptibility; points to the left decrease it. Colour encodes the feature value: blue = low, red = high. Species and drug dominate the model’s decisions in both magnitude and direction. Later isolation years (red points, year feature) consistently shift predictions upward, confirming that the model encodes the temporal resistance escalation trend. Sample type contributes minimally and without consistent directionality.

The resistance patterns in Section 3.1 represent what the model needs to learn. The following figures report how accurately it performs, starting with overall discriminative performance and moving progressively toward the granular metrics most relevant to clinical interpretation. All primary results use patient-level data splitting with no leakage across partitions. Figure 3 presents the ROC curves for both classifiers on the held-out test set and the variable importance scores from the Random Forest model.

Additional SHAP analyses including mean absolute feature importance (Supplementary Figure S7), dependence plot for the year feature by species (Supplementary Figure S8), mean SHAP contribution per antifungal drug (Supplementary Figure S9), and individual prediction waterfall plots for two representative clinical cases (Supplementary Figure S10) are provided in the Supplementary Material.

The Random Forest model achieved AUC = 0.893 (95% CI 0.852–0.930) on the patient-level test set, with group cross-validation confirming stability (0.902 ± 0.014 across five patient-stratified folds). The Logistic Regression model achieved AUC = 0.852 (95% CI 0.801–0.895). Critically, the rule-based comparator model, which simply looks up the observed species–drug non-susceptibility rate from the training data, achieved an equivalent AUC of 0.896 (95% CI 0.854–0.936). The confidence intervals of all three models overlap substantially.

The clinical value of the model is most evident when considering scenarios where intrinsic susceptibility rules do not apply, such as *C. albicans* and *C. tropicalis*, where resistance is acquired rather than intrinsic. A sensitivity analysis restricted to these two species (hard subset, *n* = 916 observations) showed RF AUC = 0.929 (95% CI 0.894–0.960) against a rule-based AUC of 0.899, representing a meaningful 0.030 incremental gain in precisely the clinical scenarios where predictions are genuinely uncertain, and rules offer less guidance.

This finding directly addresses a central methodological concern: much of the predictive signal in this dataset derives from intrinsic and well-established species-drug susceptibility patterns (*C. krusei*–fluconazole, *C. glabrata*–fluconazole) rather than from complex learned patterns unique to machine learning. The feature importance plot (Panel B) confirms this: species identity accounts for 49.7% of the model’s decisions, and antifungal drug for 37.5%, together explaining 87.2% of discriminative ability—both deterministic for several species–drug pairs. The machine learning model’s advantage over the rule-based comparator is therefore not primarily in overall AUC, but in three specific capabilities: (1) calibrated year-specific probability estimates that track temporal resistance escalation, (2) the integration of sample type information (even if marginally informative at present), and (3) the ability to update continuously as new data are added, unlike a static lookup table. Isolation year contributed 9.1% of importance, encoding the upward resistance trend.

AUC describes ranking accuracy but not the clinical consequence of errors. The confusion matrices below quantify those consequences (Table 1).

The two error types carry very different clinical consequences. A false negative (FN, resistant isolate predicted as susceptible) means the patient receives an ineffective drug; a false positive (FP, susceptible isolate flagged as resistant) causes an unnecessary therapy change with potential additional toxicity. The Random Forest model correctly classified 313 susceptible and 106 non-susceptible (I + R) isolates, generating only seven false positives; it missed 43 resistant isolates (false negatives). The Logistic Regression model detected slightly more resistant isolates (113 true positives) but generated ten times as many false positives (71 FP). The RF precision of 93.8%—meaning the model is right nearly 19 times out of 20 when it flags non-susceptibility—is the key practical advantage.

**Figure 4:**
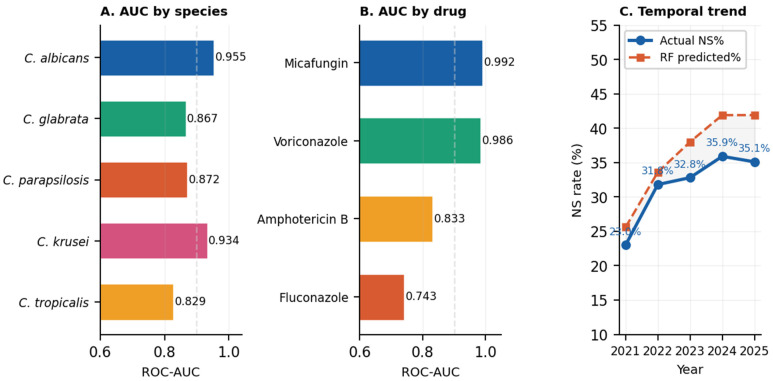
Stratified model performance and temporal trend. (**A**) Per-species ROC-AUC of the Random Forest model, computed separately for each main *Candida* species. (**B**) Per-drug ROC-AUC, computed separately for each antifungal drug. (**C**) Actual observed non-susceptibility rate (blue solid) versus RF mean predicted probability (orange dashed) by year, 2021–2025.

Overall AUC pools performance across all species and drugs, including intrinsically determined combinations. The next figure disaggregates this to identify where the model is most and least reliable. Figure 4 shows per-species and per-drug stratified AUC values, and the temporal trend of observed versus predicted non-susceptibility rates across 2021–2025.

*C. albicans* achieved the highest per-species AUC (0.955) and *C. tropicalis* the lowest (0.826). The near-perfect AUC for micafungin and voriconazole (≥0.986) is driven largely by EUCAST v12.1 breakpoint structure creating near-deterministic classification for *C. albicans*—confirming that a portion of the apparent model performance reflects instrument-specific artefacts rather than true biological prediction. A sensitivity analysis excluding *C. albicans*–micafungin and *C. albicans*–voriconazole observations (VITEK lower detection limit artefact) yielded AUC = 0.627 (95% CI 0.526–0.719), substantially lower, confirming that these combinations disproportionately inflate overall performance metrics. Fluconazole’s lower AUC (0.743) reflects genuine heterogeneity in acquired azole resistance across the cohort. Panel C shows the model tracking the year-on-year resistance escalation from 23.0% in 2021 to 35.1% in 2025. A comprehensive summary of all model performance metrics across all validation approaches is provided in Supplementary Figure S6.

Established performance is a prerequisite. The following section proposes how a probability table and a workflow could be structured pending external validation.

### 3.3. Proposed Clinical Application: A Research-Stage Decision Support Framework

**Figure 5:**
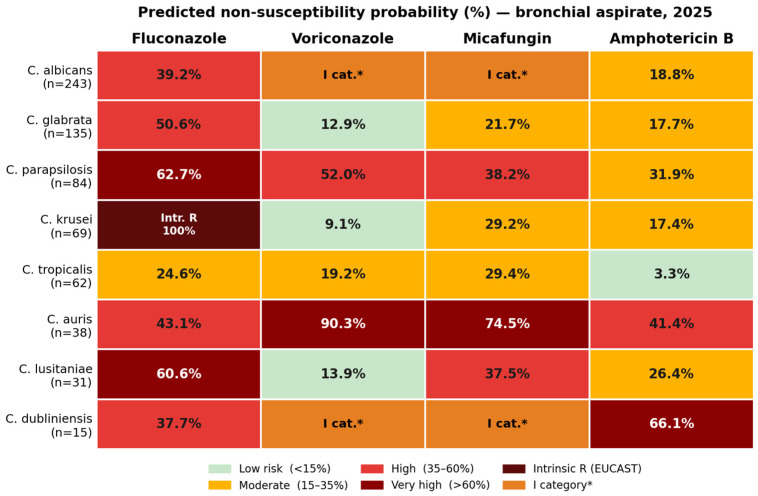
Predicted probability of antifungal non-susceptibility (%) per species × drug combination. Conditions: bronchial aspirate/sputum, isolation year 2025. * I cat. = Susceptible, Increased Exposure—not acquired resistance. Intr. R = intrinsic resistance per EUCAST. Using this table, a physician can immediately see that for *C. tropicalis* fluconazole carries a 24.6% predicted NS probability (moderate risk—reasonable to wait for the result before committing), voriconazole 19.2% (moderate), micafungin 29.4% (moderate–high), and amphotericin B just 3.3% (low risk—safest empirical option if treatment cannot be delayed). For *C. tropicalis*—a species without intrinsic resistance designations for any of these agents—this represents genuine probabilistic guidance unavailable from standard species-based rules alone.

Predictive accuracy is necessary but not sufficient for clinical utility. The following two figures illustrate how the model’s predictions could be operationalised as a reference table and embedded in a proposed ICU workflow, pending external validation. Predictions should be understood as probabilistic guidance for genuinely uncertain species–drug combinations—not as a re-encoding of intrinsic resistance rules, which are already codified in species-based stewardship guidelines. Figure 5 translates these model outputs into a probability reference table for the year 2025.

**Figure 6:**
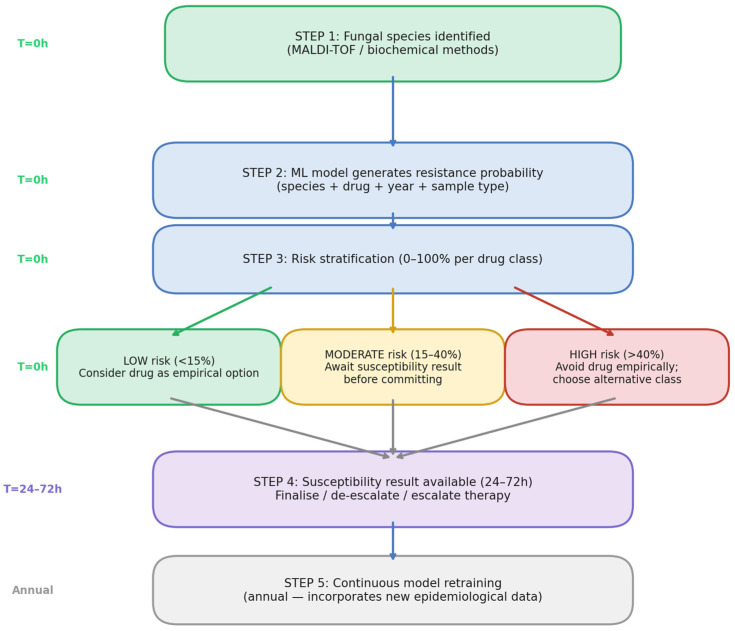
Proposed five-step ML-assisted antifungal stewardship workflow. Steps 1–3 (T = 0 h) occur simultaneously with species identification. Step 4 (T = 24–72 h) integrates definitive MIC results. Step 5 ensures annual model retraining to maintain epidemiological calibration. The workflow is proposed as a complement to—not a replacement of—standard infectious disease physician consultation and local antibiogram guidance.

For *C. auris*, predicted NS reaches 90.3% for voriconazole and 74.5% for micafungin. Complete susceptibility profiling is mandatory before any therapeutic decision. The probability table is most useful when embedded in a structured workflow. Figure 6 proposes one.

The workflow positions the model as a bridge during the 24–72 h gap between species identification and MIC availability—not as a replacement for susceptibility testing or specialist consultation. This interval carries measurable mortality consequences: inappropriate empirical antifungal choice in invasive candidiasis increases ICU mortality by 20–40% [21,22]. Annual retraining is integral to the workflow. A model trained exclusively on 2021–2023 data would substantially underestimate *C. auris*-related resistance risk in 2025; the epidemiological landscape does not stay still, and neither can the model. Prospective deployment studies comparing model-guided versus standard empirical choices are needed to validate clinical impact.

Practical implementation would require integration at three levels. At the laboratory information system (LIS) level, an automated trigger would generate model output at the moment of species entry, without requiring additional clinician input. At the antimicrobial stewardship programme level, predicted resistance probabilities would be routed to the stewardship pharmacist or infectious disease physician as part of the existing alert workflow, reviewed before any therapy modification. At the governance level, the model would require classification as a clinical decision support tool under applicable regulatory frameworks, periodic performance auditing, and documented annual retraining. Key implementation barriers include: LIS vendor API availability and integration cost; clinician training to correctly interpret calibrated probabilities rather than binary predictions; the risk of automation bias, where clinicians over-rely on model output without independent clinical assessment; and the absence of a validated governance framework for ML-based microbiological decision support in most European institutions. These barriers are surmountable but require prospective planning before any deployment attempt.

### 3.4. Advanced Machine Learning and Sensitivity Analyses

**Figure 7:**
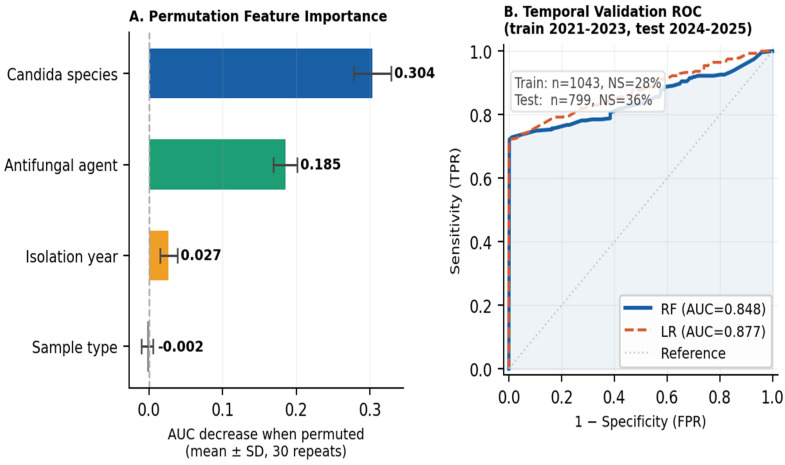
Permutation feature importance and temporal validation. (**A**) Permutation feature importance on the patient-level held-out test set: mean AUC decrease (bar) and SD across 30 permutation repeats (error bars). A bar at or below zero means the feature does not improve generalisation on unseen data. (**B**) ROC curves for pseudo-prospective temporal validation: both models trained exclusively on 2021–2023 isolates and evaluated on 2024–2025 isolates from the same institution.

Six complementary analyses address whether features are genuinely informative, whether the model holds up on future data, whether three-category prediction is feasible, whether probabilities are numerically trustworthy, and whether the results are robust to the VITEK-2 artefact and intrinsic resistance confounding. Figure 7 presents permutation feature importance on the test set and the ROC curves from the pseudo-prospective temporal validation.

Permutation importance (Panel A) confirms that species identity is by far the most informative variable (mean AUC decrease 0.304 across 30 repeats). Antifungal drug follows (0.185). Isolation year adds a real but modest contribution (0.027), encoding the upward resistance trend. Sample type showed marginally negative permutation importance (−0.002), indicating that its current five-category encoding adds no reliable signal to unseen data. Given this result, sample type should be considered for removal from future model iterations unless a finer-grained encoding can be validated. It was retained in the primary model for transparency.

Temporal validation (Panel B) mirrors prospective deployment: the model is trained on historical data and tested on genuine future cases collected when resistance prevalence was higher. Both classifiers held up—RF AUC 0.848, and LR AUC 0.877 on 2024–2025 data—despite a 7.2 percentage-point increase in resistance prevalence. This is referred to as pseudo-prospective validation, given that both periods derive from the same institution with identical testing infrastructure; true external validation at an independent centre remains to be performed. The LR outperforming RF on temporal data argues for parallel deployment of both models, with LR as a stability check when epidemiology is shifting rapidly.

The binary model pools I and R into a single non-susceptible category. That is a clinical simplification worth examining more closely.

**Figure 8:**
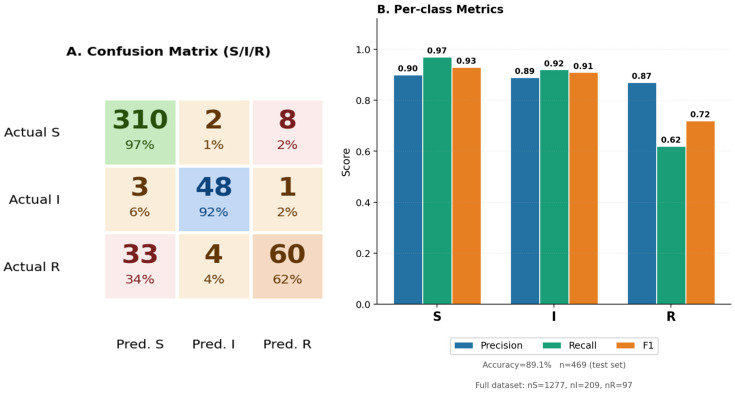
Three-category prediction (S/I/R). (**A**) 3 × 3 confusion matrix for the three-class Random Forest model on the test set (*n* = 469). Diagonal cells represent correct predictions; off-diagonal cells represent misclassifications. (**B**) Per-class precision, recall, and F1-score for each EUCAST category.

Standard binary prediction collapses the I and R categories into a single non-susceptible class, obscuring a clinically important distinction. The following analysis evaluates whether the model can reliably differentiate all three EUCAST categories. Figure 8 shows the confusion matrix and per-class precision, recall, and F1-score for the three-class S/I/Rmodel.

The three-class model achieved 89.1% overall accuracy. Performance was strong for the S category (F1 = 0.931, recall = 94.4%) and the I category (F1 = 0.906, recall = 92.3%), confirming that isolates likely to respond to dose-optimised therapy are distinguished from both truly susceptible and resistant ones. The R category showed lower recall (61.9%), with 37 of 97 truly resistant isolates misclassified: 33 predicted as S and 4 as I. These false negatives represent the most clinically consequential error type and are a firm argument against deploying the model as a standalone substitute for susceptibility testing. The model is best positioned as a triage and flagging tool, not a definitive test.

Beyond discrimination, the clinical value of probability estimates depends on their numerical accuracy. The following analysis assesses whether the model’s predicted probabilities correspond to observed resistance rates—a property known as calibration. Calibration analysis and Brier score comparison are presented in Supplementary Figure S5.

The Random Forest Brier score of 0.093 represents a 57.5% improvement over a naive classifier that ignores all four features and simply predicts the marginal prevalence (0.219). In simple terms: the model’s probability estimates carry real information. The calibration curve shows good alignment at low-to-moderate predicted probabilities, with slight underestimation in the 0.5–0.8 range. For clinical deployment, post hoc calibration using Platt scaling or isotonic regression is recommended to correct this residual bias in the range where therapeutic decisions are most uncertain.

The summary brings all evaluations into a single view. The Random Forest model is the preferred classifier for clinical deployment, demonstrating the best calibration (Brier 0.093) and highest precision (93.8%). The Logistic Regression model, though less discriminating on the random split, generalises more robustly to future data and should be run in parallel as a stability check. All models need annual retraining; performance against observed non-susceptibility rates should be monitored continuously rather than assumed to remain stable.

The following section shows how different these results are in the European landscape, by placing local resistance rates alongside the best available continental reference data.

### 3.5. European Benchmarking

**Figure 9:**
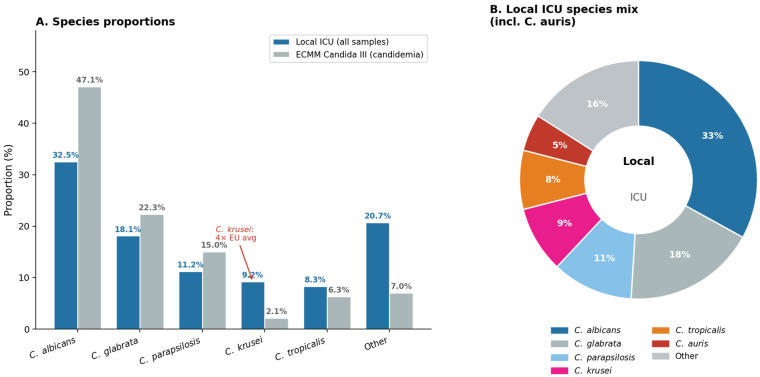
Species proportions: Local ICU vs. Europe. (**A**) Species proportions in the local ICU (blue bars, all sample types, 2021–2026) versus ECMM *Candida* III (grey bars, candidemia only, 17 countries, 2018–2022, *n* = 399). (**B**) Local ICU species distribution for 2025, including *C. auris* as a visible segment. Source: [14].

The model’s predictions are grounded in local epidemiology. Before those predictions can inform clinical practice, it is worth asking how far that local epidemiology departs from what the rest of Europe sees—and therefore how dangerous it would be to rely on continental guidelines calibrated to a different resistance landscape. To answer this, local resistance rates were compared against the ECMM *Candida* III Study, currently the most comprehensive pan-European *Candida* resistance dataset. Because the datasets differ in sample type, MIC methodology, breakpoint version, and study period, all comparisons are directional and descriptive rather than inferential. Figure 9 compares local ICU species proportions against the ECMM *Candida* III European reference dataset.

Two differences stand out immediately. *C. krusei* was found in 9.2% of local isolates —nearly four times the ECMM European average of 2.3%. Since *C. krusei* is intrinsically resistant to fluconazole per EUCAST, this means that more than one in ten ICU *Candida* isolates automatically eliminates azole therapy as an option, regardless of any susceptibility result. The machine learning model already reflects this: it assigns near-100% non-susceptibility probability to every *C. krusei*–fluconazole combination in its probability table. *C. auris*, entirely absent from the 2018–2022 ECMM dataset, reached 5.1% of local isolates overall and climbed to 14% of annual isolates by 2025—a trajectory that the ECMM data, collected before the Eastern European outbreak, could not have anticipated.

**Figure 10:**
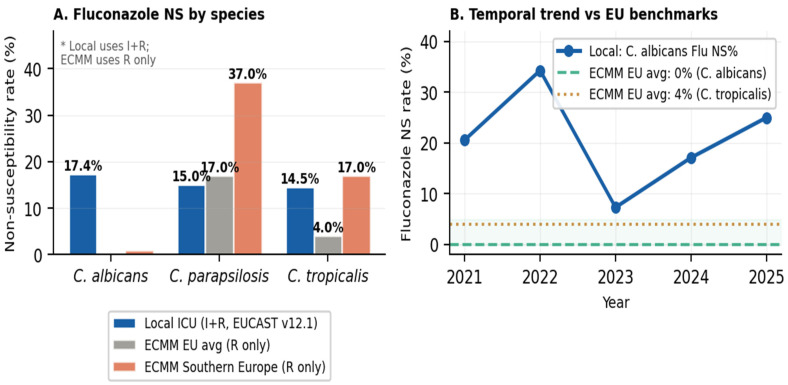
Fluconazole resistance: Local ICU vs. European benchmarks. (**A**) Fluconazole non-susceptibility for three species: local ICU (blue, I + R per EUCAST v12.1), ECMM EU average (grey, R only), and ECMM Southern European subset—Greece, Italy, Turkey (orange, R only). (**B**) Year-by-year *C. albicans* fluconazole NS trajectory versus ECMM EU benchmarks. Sources: [14,17].

Species mix shapes the resistance risk; drug-level resistance rates determine the magnitude of that risk for each therapeutic choice. Figure 10 shows fluconazole non-susceptibility rates for the three most affected species against European benchmarks.

**Figure 11:**
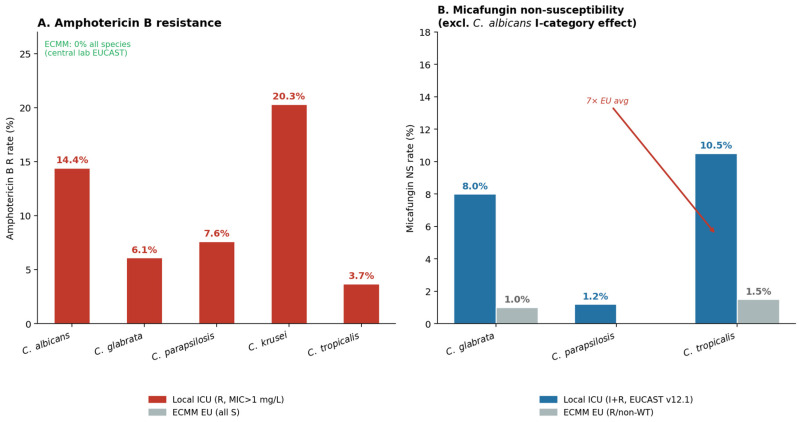
Amphotericin B and micafungin non-susceptibility rates versus European reference data (**A**) Amphotericin B resistance rates per *Candida* species: local ICU (automated VITEK-2 testing, MIC > 1 mg/L classified as R per EUCAST) versus ECMM *Candida* III European reference (0% for all species by central reference laboratory EUCAST broth microdilution). (**B**) Micafungin non-susceptibility (I + R per EUCAST v12.1) for three species (*C. glabrata*, *C. parapsilosis*, and *C. tropicalis*); *C. albicans* excluded as voriconazole and micafungin I-category designations reflect instrument detection limits rather than acquired resistance. ECMM source: [23].

The ECMM *Candida* III Study reported zero fluconazole resistance in *C. albicans* across all 17 participating countries. Our local data, applying an equivalent R-only threshold (MIC ≥ 8 mg/L), yield approximately 16.1%—a difference far too large to be explained by breakpoint version alone. The bimodal MIC distribution in Supplementary Figure S2 provides direct microbiological confirmation of genuine acquired resistance. For *C. tropicalis*, the local NS rate (14.5%) is nearly four times the ECMM European average of 4%, a pattern consistent with elevated azole resistance reported across Eastern Europe and the Mediterranean. *C. parapsilosis* (15.0%) falls within the European range and is the one species for which this ICU is not an outlier. The conclusion is straightforward: fluconazole empirical therapy guidelines calibrated to Western European data—where *C. albicans* azole resistance is effectively zero—are not applicable to this setting. Figure 11 compares local amphotericin B and micafungin resistance rates against ECMM European reference data.

The ECMM study found zero amphotericin B resistance across all species; our automated testing system identified 11.4% resistance overall. This gap is almost certainly larger than true biology can explain alone. Automated susceptibility systems are known to systematically overestimate MIC values for polyenes relative to reference broth microdilution, and that artefact likely accounts for a meaningful fraction of the apparent discrepancy [24]. Reference laboratory confirmation of a representative subsample is recommended before any clinical conclusions are drawn about true local amphotericin B resistance. The *C. tropicalis* micafungin finding is more straightforward: 10.5% non-susceptibility locally against approximately 1.5% in ECMM is a robust signal. Echinocandin resistance in *C. tropicalis* is mediated by FKS1/FKS2 mutations [23,25,26], is associated with treatment failure, and if confirmed by reference methodology would require routine FKS gene sequencing for this species.

The following summary table (Table 2) places all comparisons side by side.

The pattern is consistent: local rates exceed ECMM benchmarks for every metric except *C. parapsilosis* fluconazole, which falls within the European range. This is not an isolated finding—it reflects a well-documented Eastern European epidemiological profile, with higher non-albicans *Candida* prevalence, higher azole resistance in *C. albicans* and *C. tropicalis*, and now accelerating *C. auris* emergence. The implication for antifungal stewardship is direct: a clinician in Bucharest in 2025 who relies on pan-European guidelines to guide empirical therapy might need additional help from local guidelines and tools, such as this machine learning model.

## 4. Discussion

Five years of microbiological data from a single ICU have produced three findings that clinicians and microbiologists in Eastern European centres will recognise and find concerning. The first is the MDR trajectory (Supplementary Figure S1). Resistance to multiple antifungal classes simultaneously has moved from one in four isolates in 2021 to one in two in 2025. A physician prescribing fluconazole empirically for an unidentified *Candida* infection in our ICU in 2025, without susceptibility data, has less than a 50% chance of choosing an appropriate drug. This is not a gradual drift—it is an acceleration. The second finding is *C. auris*. Absent in 2021, it reached 16 isolates per year by 2025 and accounted for 14% of annual isolates [24,27]. *C. auris* is resistant to fluconazole in over 90% of strains globally, can survive on hospital surfaces for weeks, spreads between patients who are physically separated, and is routinely misidentified by standard biochemical methods [24,27]. Romania has been identified as one of the five European countries with the highest *C. auris* burden [6,25,27]. Every new isolation in this cohort represents a potential outbreak signal that demands immediate infection control action [24,26,27]. The third finding is structural: resistance in this ICU follows learnable, species-specific patterns. Species identity and drug choice together account for 87.2% of the predictive signal [14]. That is both a statement about the epidemiology—resistance is not random, it clusters along recognisable taxonomic and pharmacological lines—and a statement about what the model can do. A prediction of 62.7% fluconazole non-susceptibility for a *C. parapsilosis* blood culture isolate in 2025 is not a guess; it is a quantified probability derived from five years of local data, available within seconds of the species report.

A well-maintained local antibiogram delivers comparable aggregate discrimination—this finding itself argues for mandatory local surveillance in Eastern European ICUs where pan-European benchmarks are demonstrably inapplicable. The ML model’s value is in automating temporal recalibration and providing probability gradients for acquired resistance scenarios, where a static table cannot differentiate 2021 from 2025.

The benchmarking data make the argument directly: *C. albicans* fluconazole resistance runs at approximately 16% locally against 0% in the ECMM dataset; *C. krusei* is four times more prevalent than the European average. A clinician who reaches for fluconazole empirically following guidelines calibrated to Western European data is working from a resistance map that does not reflect this patient population [18,22,23]. The machine learning model encodes local resistance knowledge at the moment of decision, though the majority of this signal derives from established species–drug patterns rather than genuinely learned biological complexity. As the rule-based comparator analysis confirms, a significant share of that value can also be delivered by a well-maintained local antibiogram table; the machine learning model’s specific incremental contributions—temporal calibration, probability quantification, and meaningful prediction for species without intrinsic resistance designations—are detailed in the following subsection.

The rule-based comparator model—which simply applies the empirical species–drug non-susceptibility rate observed in the training data, without incorporating year or sample type—achieved AUC = 0.896 (95% CI 0.854–0.936), statistically indistinguishable from the Random Forest (AUC = 0.893, 95% CI 0.852–0.930) model. This finding must be stated clearly: the majority of the aggregate predictive performance in this dataset derives from intrinsic and established species-drug susceptibility patterns already known to clinicians and encoded in guidelines. A species-aware local antibiogram provides comparable overall discrimination.

The machine learning model adds value over a static lookup table in three specific ways. First, it provides year-specific probability calibration that tracks temporal resistance escalation without manual updating—the year feature contributed 9.1% of importance. Second, it demonstrates meaningful incremental gains in genuinely uncertain predictions: for *C. albicans* and *C. tropicalis*—species without intrinsic resistance designations where rules offer less clear guidance—the hard-subset analysis showed RF AUC = 0.929 versus rule-based AUC = 0.899, a 0.030 gain corresponding to clinically real uncertainty. Third, it is structurally ready to incorporate additional features (prior antifungal exposure, patient risk factors) as these become available, unlike a static table.

The binary non-susceptibility target (I + R) was chosen to align with clinical practice: I-category isolates may require dose optimisation or alternative agents rather than guaranteed standard response. However, this definition introduces a documented artefact for *C. albicans* voriconazole and micafungin. The VITEK-2 lower detection limit (≤20.06 mg/L for micafungin; ≤20.12 mg/L for voriconazole) exceeds the EUCAST v12.1 S breakpoints, causing all *C. albicans* isolates tested with these drugs to be assigned to category I regardless of their true MIC. The sensitivity analysis excluding these combinations yielded AUC = 0.627 (95% CI 0.526–0.719), confirming that their inclusion in the primary analysis substantially inflates apparent performance. The R-only sensitivity analysis—restricting the target to confirmed acquired resistance—yielded RF AUC = 0.861 (95% CI 0.806–0.909), representing a more conservative and methodologically robust performance estimate. Future model iterations should consider excluding instrument-truncated combinations from training or treating them as a separate prediction task.

Patient-level Group K-Fold cross-validation (AUC 0.902 ± 0.014) confirmed that the primary performance estimates are not inflated by patient-level data leakage. Permutation importance confirmed species and drug as genuine, independent predictors; the negative permutation importance for sample type (−0.002) argues for its removal or re-encoding in future iterations. Temporal pseudo-prospective validation demonstrated useful discrimination on 2024–2025 data—this is pseudo-prospective because both periods derive from the same institution with the same testing infrastructure, not external validation. The three-class analysis showed reliable S and I identification but lower R recall (61.9%), reinforcing that the model is a triage and flagging tool rather than a definitive test. Calibration analysis confirmed numerically reliable RF probability estimates (Brier 0.093, 57.5% improvement over naïve baseline), with post hoc calibration recommended for the 0.5–0.8 probability range. Taken together, these validation results confirm that the model performs reliably within the institution where it was developed. They do not constitute evidence of generalizability. External prospective validation at independent centres with different patient populations, species distributions, and testing infrastructures is a prerequisite before clinical implementation—not a recommended next step, but a mandatory one.

The 10.5% micafungin non-susceptibility in *C. tropicalis*—against approximately 1.5% in the ECMM *Candida* III dataset—warrants separate discussion. Echinocandin resistance in *C. tropicalis* is caused by FKS1 hotspot point mutations [28,29,30] and reliably predicts clinical treatment failure. If this rate is confirmed by reference broth microdilution, routine FKS1 sequencing for *C. tropicalis* isolates may be justified in this setting, and echinocandin therapy for this species without susceptibility testing would carry meaningful failure risk. Molecular confirmation is needed before definitive recommendations can be made.

In order to improve the model, several steps would be beneficial for the surveillance programme and the model. First, *C. auris* surveillance needs to be uniformed: MALDI-TOF or molecular species confirmation of all suspected isolates, complete susceptibility profiling, and automatic infection control notification [24,26]. Second, flucytosine testing should become routine for *C. glabrata*, *C. tropicalis*, and sterile-site isolates; 29.6% coverage is inadequate surveillance. Third, a representative subsample of automated-system “resistant” amphotericin B isolates should be sent to a reference laboratory for EUCAST broth microdilution confirmation [23]. Fourth, a pilot LIS integration should be built, with prospective tracking of model predictions against observed MIC outcomes and formal evaluation of whether model-guided empirical choices differ from standard care [31]. Fifth, prior antifungal exposure should be incorporated as a feature—the individual patient-level factor most consistently associated with acquired resistance [21,22]—even as a binary variable. Beyond antifungal exposure, additional clinical variables that could meaningfully enhance future model versions include: prior *Candida* colonisation status, particularly relevant for *C. auris* and fluconazole-resistant *C. parapsilosis* where colonisation pressure precedes infection; duration of ICU stay at the time of isolation, as prolonged hospitalisation increases cumulative antifungal selection pressure; immunosuppression status—including corticosteroid therapy, haematological malignancy, and solid organ transplant—which modulates both the probability of invasive infection and the species distribution encountered; and recent broad-spectrum antibacterial exposure, which promotes *Candida* overgrowth and indirectly increases resistance selection pressure. Each of these variables is routinely documented in ICU electronic health records and could be extracted without additional data collection infrastructure.

An important limitation applies specifically to respiratory isolates, which represent 52.8% of this cohort. Bronchial aspirate and sputum specimens encompass both *Candida* colonisation—which is common in ICU patients receiving broad-spectrum antibiotics and does not require antifungal treatment—and genuine invasive pulmonary candidiasis, which is rare and diagnostically challenging. Because the distinction between these two entities was not systematically recorded in the laboratory information system, the model was trained on a mixture of colonising and invasive isolates without this information being available as a feature. For bloodstream isolates (5.1%) and sterile-site specimens (approximately 10%), clinical significance is unambiguous and model predictions are more directly actionable. For respiratory isolates, the predicted resistance probability should be interpreted as a property of the organism if treatment were to be initiated—not as a recommendation to treat. The decision to treat a respiratory *Candida* isolate remains a clinical judgement that precedes and is independent of resistance prediction.

## 5. Limitations

Single-centre, retrospective design limits generalizability; the model should be retrained and locally validated before application in any other setting. Based on the training set composition used in this study—1408 isolate–drug observations from 437 patients across five principal species and six antifungals—a minimum of approximately 400–500 patient-level isolate-drug observations is recommended as a practical threshold for meaningful local retraining, covering at least four to five species and four antifungals. Below this threshold, class imbalance becomes difficult to manage and patient-level GroupShuffleSplit validation may be unstable. Centres with highly skewed species distributions—for example, those experiencing endemic *C. auris*—may require larger datasets or species-specific oversampling strategies. The Logistic Regression model is preferred over Random Forest for retraining at smaller centres given its greater stability under limited sample sizes. The feature set excludes prior antifungal exposure, likely the most important individual patient driver of acquired resistance [2]. Patient-level data splitting was applied to eliminate leakage, but the pseudo-prospective temporal validation is not equivalent to external validation. The VITEK-2 instrument artefact for *C. albicans* voriconazole and micafungin inflates primary AUC metrics; the sensitivity analyses provide more conservative estimates. *C. auris* is substantially undertested, limiting model predictions for this species. Flucytosine at 29.6% testing coverage remains incompletely characterised. Respiratory specimens predominate (52.8%), encompassing both colonisation and invasive infection; this distinction was not systematically recorded and represents a substantive limitation on the clinical applicability of model predictions for bronchial and sputum isolates specifically. Clinicians must independently determine whether treatment is indicated before applying resistance probability estimates. All susceptibility testing was performed on the VITEK-2 automated system (bioMérieux). Internal quality control was performed weekly for both species identification and antifungal susceptibility testing using EUCAST-recommended reference strains. External quality control was conducted twice yearly through the national external quality assessment programme. External reference laboratory confirmation by EUCAST broth microdilution is currently underway as part of a separate prospective study.

Directional feature attribution was not included in the primary analysis; variable importance in Figure 3B reflects mean decrease in node impurity, which quantifies the magnitude but not the direction of each feature’s contribution. SHAP analysis has been performed as a complementary analysis and results are presented in Figure 3C and Supplementary Figures S7–S10.

## 6. Conclusions

Antifungal resistance in this ICU has evolved to a degree that makes empirical therapy without local data untenable. The local epidemiological profile diverges substantially from European benchmarks, with *C. auris* now representing a significant and growing proportion of annual isolates. A machine learning model trained on local microbiological data may provide calibrated resistance probability estimates at species identification, before susceptibility results are available. Performance is comparable to a local species-drug lookup table in aggregate, with incremental advantage for species where resistance is acquired rather than intrinsic. Methodological limitations—including instrument-specific classification artefacts and the absence of patient-level predictors—require resolution, and external prospective validation at independent centres is a prerequisite before clinical implementation. The resistance landscape documented here is itself clinically actionable, independently of the model’s future development.

## Figures and Tables

**Table 1:** Confusion matrices and performance metrics. Evaluated on the held-out test set (*n* = 469 isolate–drug observations). NS = non-susceptible (I + R per EUCAST v12.1). (**A**) Confusion matrix for Random Forest on the patient-level test set (*n* = 465). (**B**) Confusion matrix for Logistic Regression. (**C**) Performance summary. TN = true negative (susceptible correctly predicted); TP = true positive (resistant correctly identified); FP = false positive (susceptible isolate incorrectly flagged as resistant—false alarm); FN = false negative (resistant isolate missed—the clinically more dangerous error type).

**A. Random Forest**		
	**Predicted S**	**Predicted NS**
Actual S	313 *True S*	7 *False NS*
Actual NS	43 *False S*	106 *True NS*
Accuracy = 89.3% | *n* = 469 test isolates | NS = non-susceptible (I + R per EUCAST v12.1)		
**B. Logistic Regression**		
	**Predicted S**	**Predicted NS**
Actual S	249 *True S*	71 *False NS*
Actual NS	36 *False S*	113 *True NS*
Accuracy = 77.2% | *n* = 469 test isolates | NS = non-susceptible (I + R per EUCAST v12.1)		
**C. Model Performance Summary**		
**Metric**	**Random Forest**	**Logistic Regression**
AUC (test set)	0.888	0.853
CV AUC (5-fold)	0.911 ± 0.008	0.865 ± 0.017
Accuracy	89.3%	77.2%
Precision (NS class)	93.8%	61.4%
Recall (NS class)	71.1%	75.8%
F1-score (NS class)	0.809	0.679
NS = non-susceptible (I + R per EUCAST v12.1). CV = cross-validation. AUC = area under the ROC curve.		

**Table 2:** Summary benchmarking table—Local ICU vs. ECMM *Candida* III European Reference. Summary comparison of all local ICU antifungal resistance metrics against the ECMM *Candida* III European multicentre reference ([23]; 399 candidemia isolates, 41 centres, 17 countries, 2018–2022, EUCAST broth microdilution in central reference laboratories). All comparisons are descriptive; formal statistical testing was not performed given methodological differences between the datasets (sample types, MIC methodology, breakpoint versions, and study periods). ↑ = local rate substantially exceeds European benchmark; ≈ = broadly comparable; — = not directly comparable due to EUCAST v12.1 breakpoint revision.

Comparison	Local ICU	ECMM EU Reference	Direction	Clinical Note
*C. albicans—Fluconazole*	~16.1% (R-only)	0% R (ECMM)	↑ Higher	Substantially above all 17 ECMM countries. Genuine acquired resistance confirmed by bimodal MIC distribution (ERG11 point mutations)
*C. glabrata—Fluconazole*	IE/WT-I	12% R (ECMM)	—	Not comparable: EUCAST v12.1 places entire wild type in category I (S ≤ 0.001 mg/L). ECMM used older breakpoints. Clinical relevance: fluconazole unreliable for *C. glabrata* regardless of interpretation
*C. parapsilosis—Fluconazole*	15.0% (I + R)	17% R (ECMM)	≈ Similar	Within European range. Southern EU (Greece, Italy, Turkey) reaches 17–37%
*C. tropicalis—Fluconazole*	14.5% (I + R)	4% R (ECMM)	↑ Higher	~3.6× EU average. Consistent with elevated azole resistance across Eastern Europe and the Mediterranean basin
*All species—Amphotericin B*	11.4% (R)	0% (ECMM)	↑ Higher	ECMM used central reference BMD; automated systems overestimate polyene MICs. Discrepancy likely partly methodological. Reference confirmation recommended before clinical conclusions
*C. tropicalis—Micafungin*	10.5% (I + R)	~1.5% (ECMM)	↑ Higher	Clinically significant. FKS1/FKS2 hotspot mutations confirmed in published series. If confirmed locally by reference BMD, would necessitate routine molecular characterisation for this species
*C. glabrata—Micafungin*	8.0% (I + R)	1% (ECMM)	↑ Higher	Warrants monitoring. Echinocandin-first empirical strategy should be supported by susceptibility data for *C. glabrata*
*C. parapsilosis—Micafungin*	1.2% (I + R)	~0% (ECMM)	≈ Similar	Consistent with European data. *C. parapsilosis* has inherently higher MICs (EUCAST breakpoint S ≤ 4 mg/L vs. S ≤ 0.03 mg/L for other species)
*C. krusei—Prevalence*	9.2% of isolates	2.3% (ECMM)	↑ 4 × higher	Distinctive local feature. Every *C. krusei* isolate is intrinsically resistant to fluconazole—automatically limits azole empirical therapy for >1 in 10 ICU isolates regardless of susceptibility testing
*C. auris—Prevalence*	5.1% overall 14% in 2025	Not reported (2018–2022)	↑ Emerging	0 isolates (2021) → 16/year (2025); 8-fold increase in 3 years. Romania among top-5 EU countries for *C. auris* burden (ECDC 2024). Not represented in ECMM *Candida* III (collected before Eastern European outbreak)

Abbreviations: I + R = non-susceptible per EUCAST v12.1 (I = Susceptible Increased Exposure, R = Resistant). R-only = resistant per EUCAST (MIC above R breakpoint). WT = wild type. IE = insufficient evidence. BMD = broth microdilution. Additional source: ECDC Survey on the Epidemiological Situation, Laboratory Capacity and Preparedness for Candidozyma (*Candida*) *auris*, 2024. Stockholm: ECDC, 2025. Local data: all biological sample types (bronchial aspirate/sputum 52.8%, urine 21.4%, blood culture 5.1%, other 20.7%); automated susceptibility testing (VITEK-2); EUCAST v12.1 reinterpretation.

## Data Availability

The original contributions presented in this study are included in the article/Supplementary Material. Further inquiries can be directed to the corresponding author.

## Supplementary Materials

The following supporting information can be downloaded at https://www.mdpi.com/article/10.3390/medsci14020319/s1. Figure S1. Multidrug Resistance Trend and *C. auris* Emergence (2021–2025); Figure S2. MIC Distributions—Evidence of Acquired Resistance; Figure S3. Co-Resistance Patterns and Non-Susceptibility by Sample Source; Figure S4. MDR Burden and Antifungal Testing Coverage. Figure S5. Calibration Analysis; Figure S6. Comprehensive Model Performance Summary; Figure S7. SHAP Feature Importance Bar Plot; Figure S8. SHAP Dependence Plot; Figure S9. SHAP Drug-Level Attribution; Figure S10. SHAP Waterfall Plots.
