# Supplementary material for: From Species Identification to Empirical Therapy: A Machine Learning and Rule-Based Decision Support Framework for Antifungal Resistance Prediction in ICU Candida Infections
Source: Med Sci (Basel). 2026 Jun 15;14(2):319. doi: 10.3390/medsci14020319 (PMC13304419; doi:10.3390/medsci14020319)
Supplement: Supplementary file 1 [file medsci-14-00319-s001.zip › medsci-4362464-supplementary.pdf]

Supplementary Material

From Species Identification to Empirical Therapy: A Machine Learning and Rule-Based Decision Support Framework for Antifungal Resistance Prediction in ICU Candida Infections

Supplementary Figure S1. Multidrug Resistance Trend and *C. auris* Emergence (2021–2025)

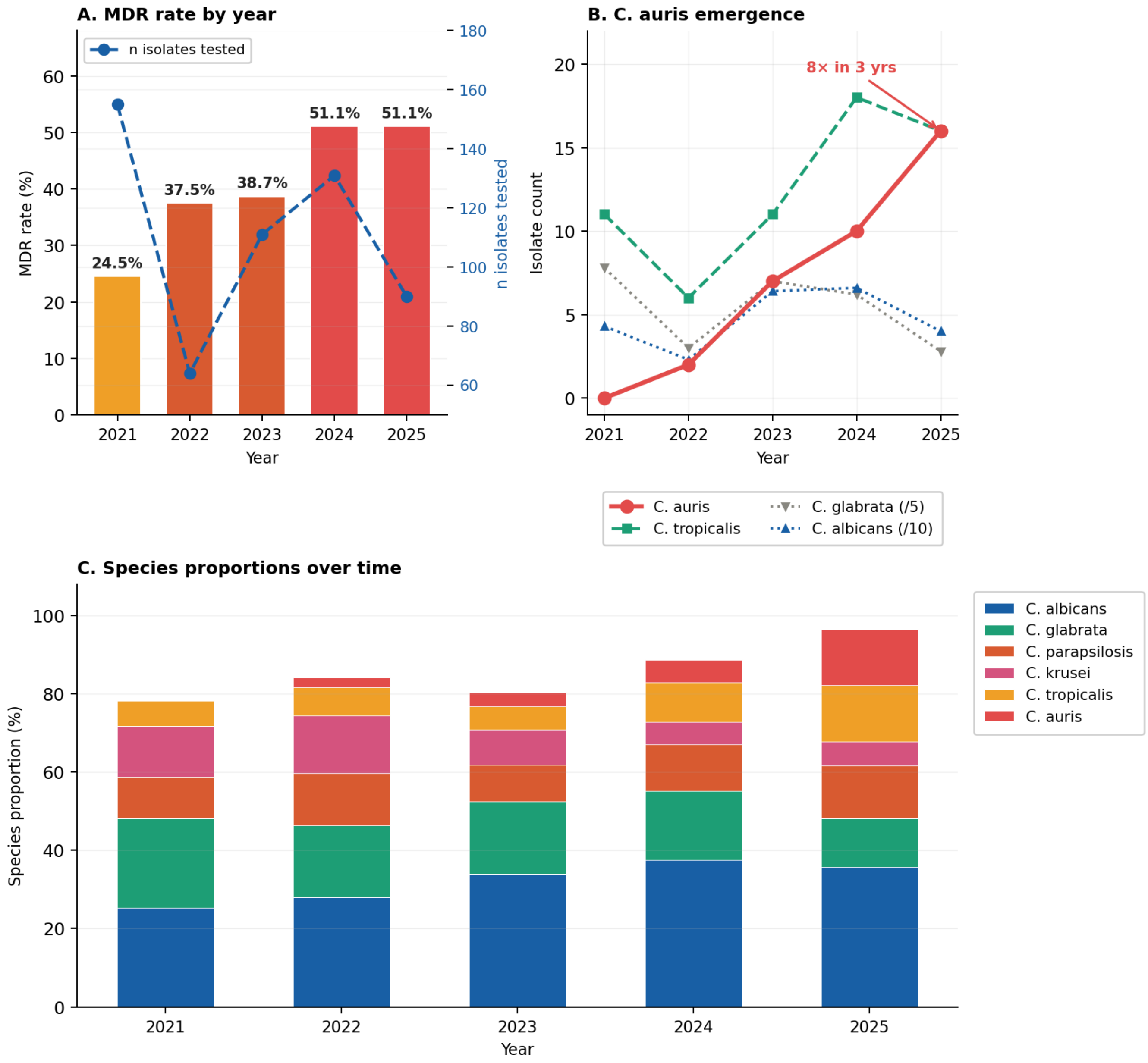

Supplementary Figure S1. (A) Annual multidrug resistance (MDR) rate, defined as non-susceptibility to two or more antifungal drug classes among those tested simultaneously per isolate. Bar colours: yellow below 35%, orange 35-50%, red above 50%. Blue line (right axis) = total isolates tested per year. (B) Annual C. auris isolate counts (solid red) versus dominant species scaled for visual comparability: C. albicans ÷10, C. glabrata ÷5, C. tropicalis. Arrow annotates the 8-fold increase in three years. (C) Relative species proportions as percentage of annual total, 2021-2025, showing C. auris as a growing red segment reaching 14% by 2025.

Supplementary Figure S2. MIC Distributions — Evidence of Acquired Resistance

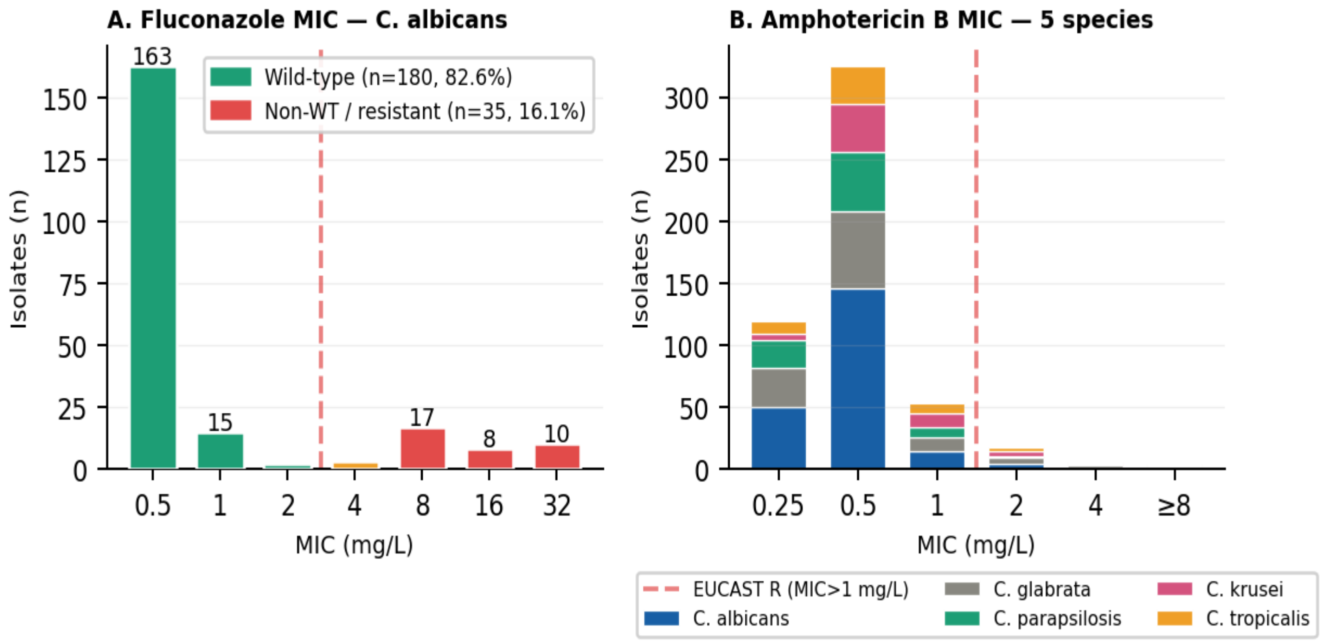

Supplementary Figure S2. (A) Fluconazole MIC distribution for C. albicans isolates with quantitative MIC results (n=218). Bars colour-coded: green for susceptible range (≤2 mg/L), yellow for the intermediate zone (4 mg/L), red and dark red for the resistant range (≥8 mg/L). Vertical dashed line marks the EUCAST S/R boundary. (B) Amphotericin B MIC distributions for five main species, stacked bars. Vertical dashed line marks the EUCAST resistance threshold (R if MIC >1 mg/L).

Supplementary Figure S3. Co-Resistance Patterns and Non-Susceptibility by Sample Source

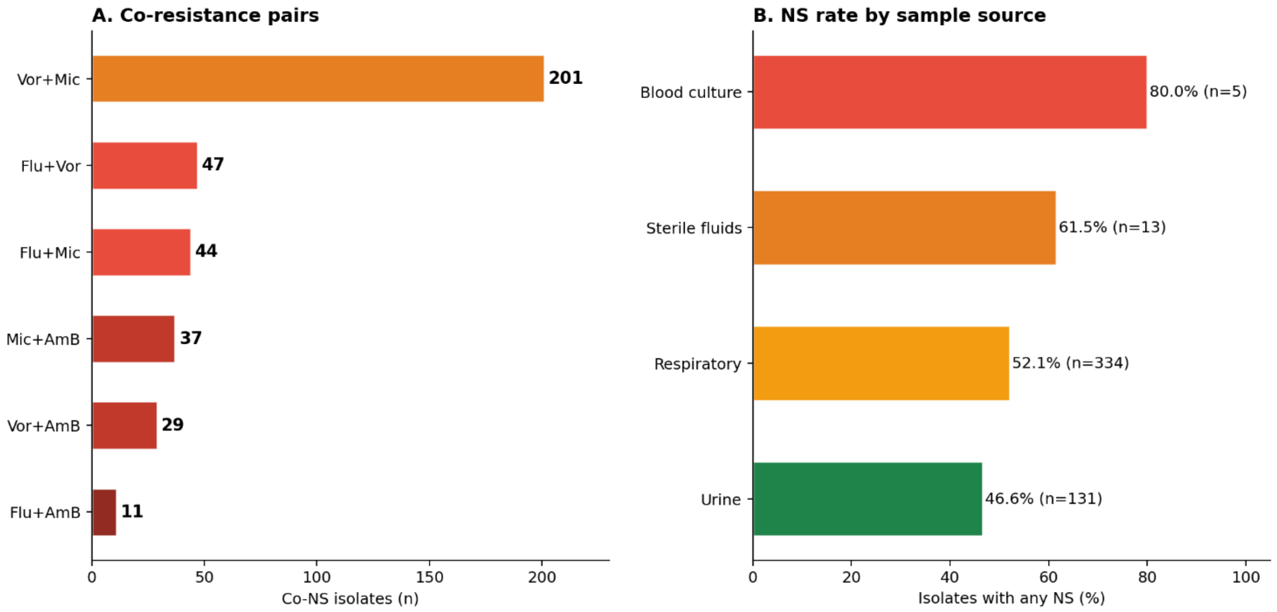

Supplementary Figure S3. (A) Number of isolates simultaneously non-susceptible to each pairwise antifungal drug combination, among isolates tested with both drugs. Vor+Mic (n=201) is predominantly driven by EUCAST v12.1 I-category reclassification of C. albicans, not acquired co-resistance. (B) Proportion of isolates with at least one non-susceptible result, stratified by biological sample type.

Supplementary Figure S4. MDR Burden and Antifungal Testing Coverage

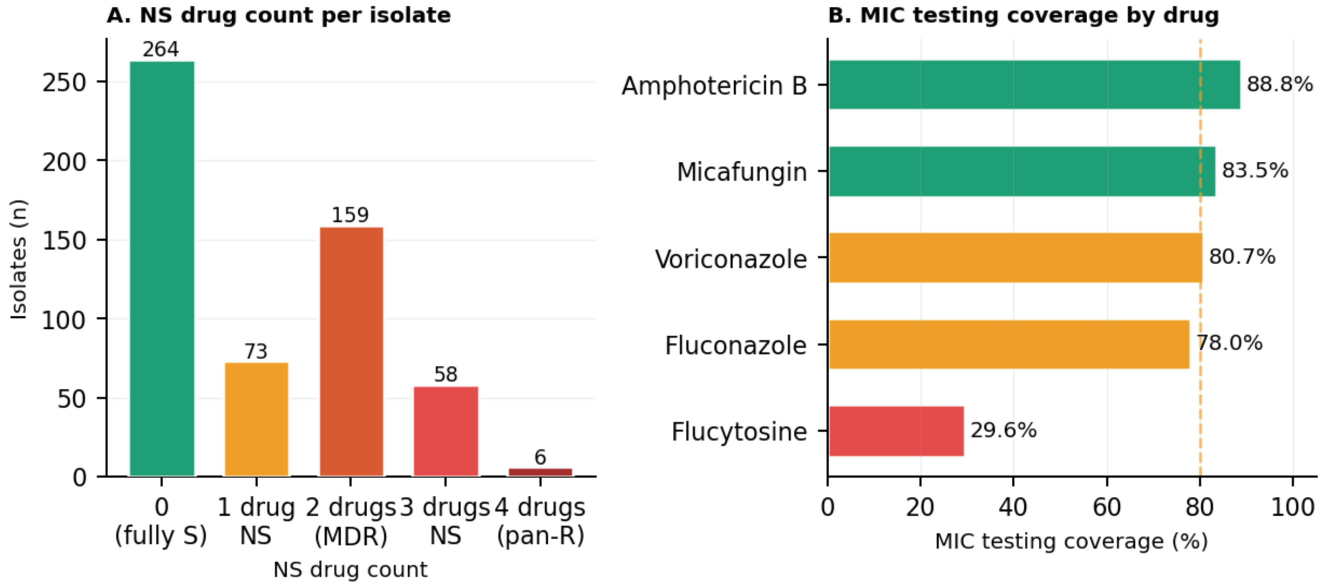

Supplementary Figure S4. (A) Distribution of 560 isolates tested with at least two antifungal drugs simultaneously, by number of antifungal drug classes to which they were non-susceptible. Colours: green = fully susceptible, amber = 1 drug NS, orange = 2 drugs (MDR), red = 3 drugs, dark red = 4 drugs (pan-resistant). (B) Proportion of the 747 total isolates with a quantitative MIC result reported, by antifungal agent. Dashed line marks 80% coverage.

Supplementary Figure S5. Calibration Analysis

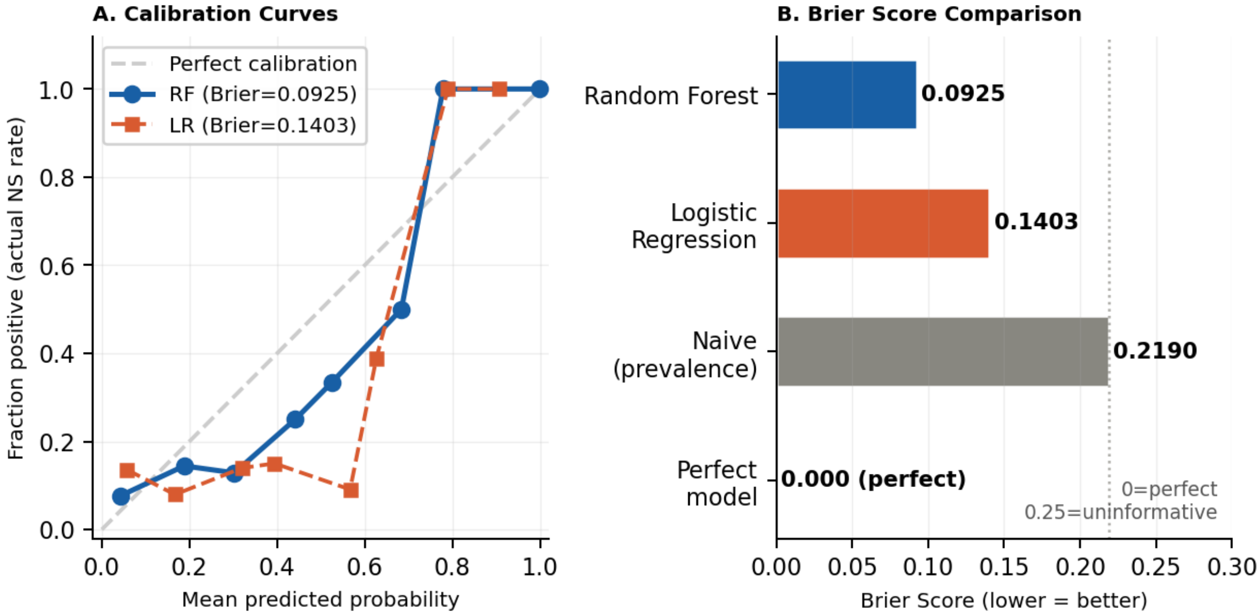

Supplementary Figure S5. Calibration analysis. (A) Calibration curves for the Random Forest (blue circles) and Logistic Regression (orange squares). The diagonal dashed line represents perfect calibration. Points above the diagonal indicate underestimation of the true resistance rate; points below indicate overestimation. (B) Brier score comparison: RF 0.093, LR 0.140, naïve classifier 0.219, perfect model 0.0. Lower scores indicate better probabilistic accuracy.

Supplementary Figure S6. Comprehensive Model Performance Summary

Analysis	Model	AUC	Accuracy	Precision (NS)	Recall (NS)	F1 (NS)	Brier
Random split (75/25)	Random Forest	**0.885**	88.5%	89.3%	72.5%	0.800	0.093
	Logistic Reg.	**0.852**	77.2%	61.4%	75.8%	0.679	0.140
Temporal split (2021-23 → 2024-25)	Random Forest	**0.848**	—	—	—	—	—
	Logistic Reg.	**0.877**	—	—	—	—	—
3-class S/I/R (random split)	S class	—	89.1%	89.6%	96.9%	0.931	—
	I class	—		88.9%	92.3%	0.906	
	R class	—		87.0%	61.9%	0.723	

NS = non-susceptible (I+R). Temporal split is the most clinically realistic validation.

Supplementary Figure S6. Summary comparison of all models and validation approaches. Green rows: standard random 75/25 split. Blue rows: temporal validation (train 2021–2023, test 2024–2025). Yellow rows: three-class model per-category performance. AUC values in bold. Dashes indicate the metric is not applicable to that analysis configuration.

Supplementary Figure S7. SHAP Feature Importance Bar Plot

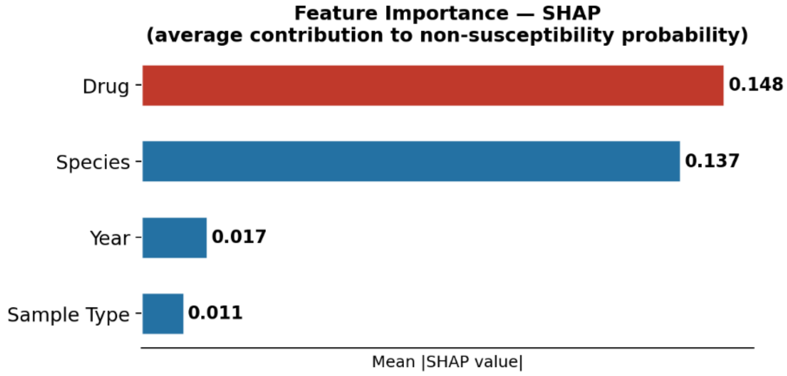

*Supplementary Figure S7***.** *SHAP Feature Importance Bar Plot. Mean absolute SHAP values for each predictor variable across the held-out test set, quantifying average magnitude of contribution to predicted non-susceptibility probability.*

Supplementary Figure S8. SHAP Dependence Plot

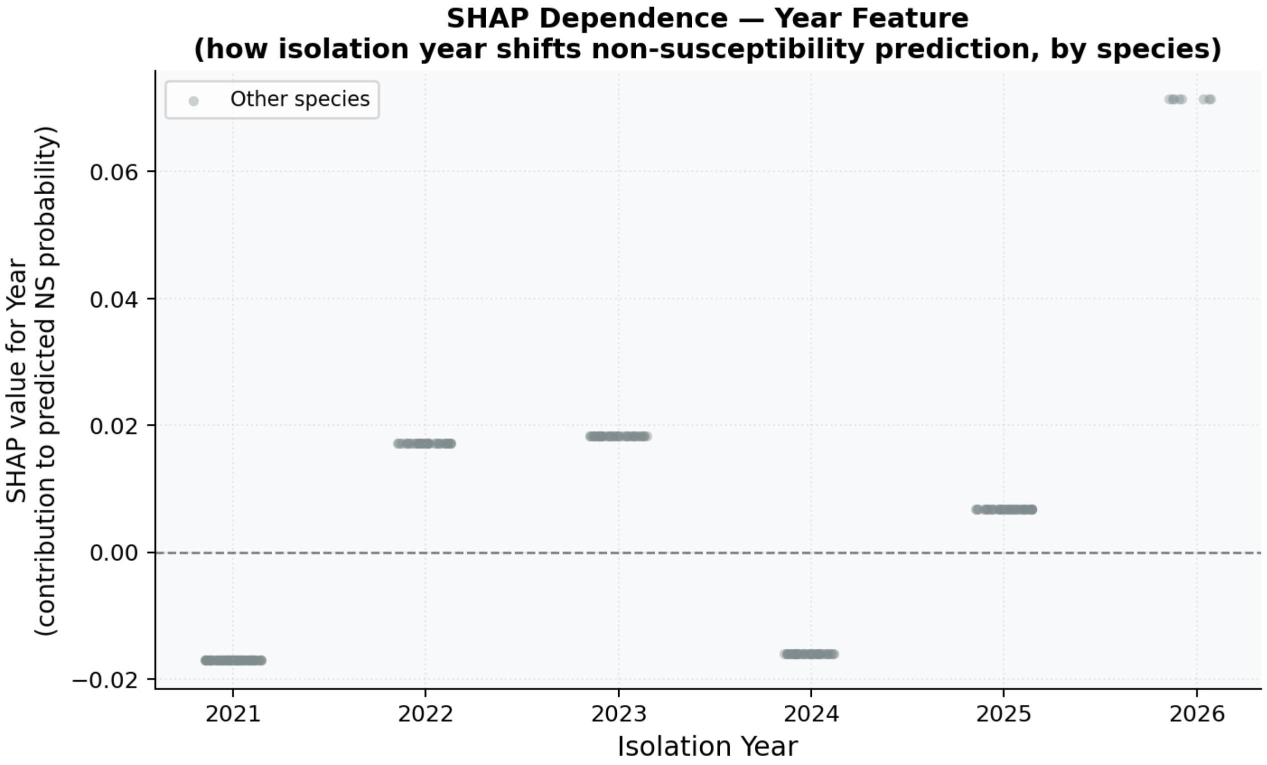

Supplementary Figure S8. SHAP Dependence Plot. Isolation Year by Species. Individual SHAP values for the Year feature plotted against isolation year, stratified by species, showing the direction and magnitude of temporal resistance trend encoding by the model.

Supplementary Figure S9. SHAP Drug-Level Attribution

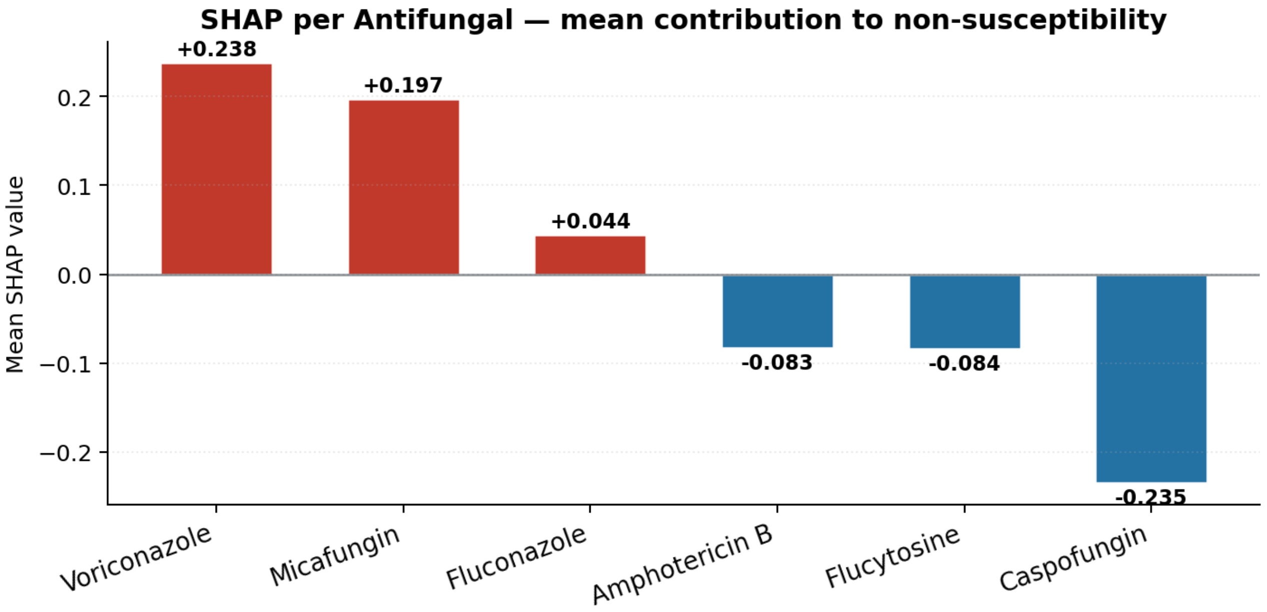

Supplementary Figure S9. SHAP Drug-Level Attribution. Mean SHAP values per antifungal drug, showing which drugs are associated with higher (positive SHAP) versus lower (negative SHAP) predicted non-susceptibility probability across the test set.

Supplementary Figure S10. SHAP Waterfall Plots

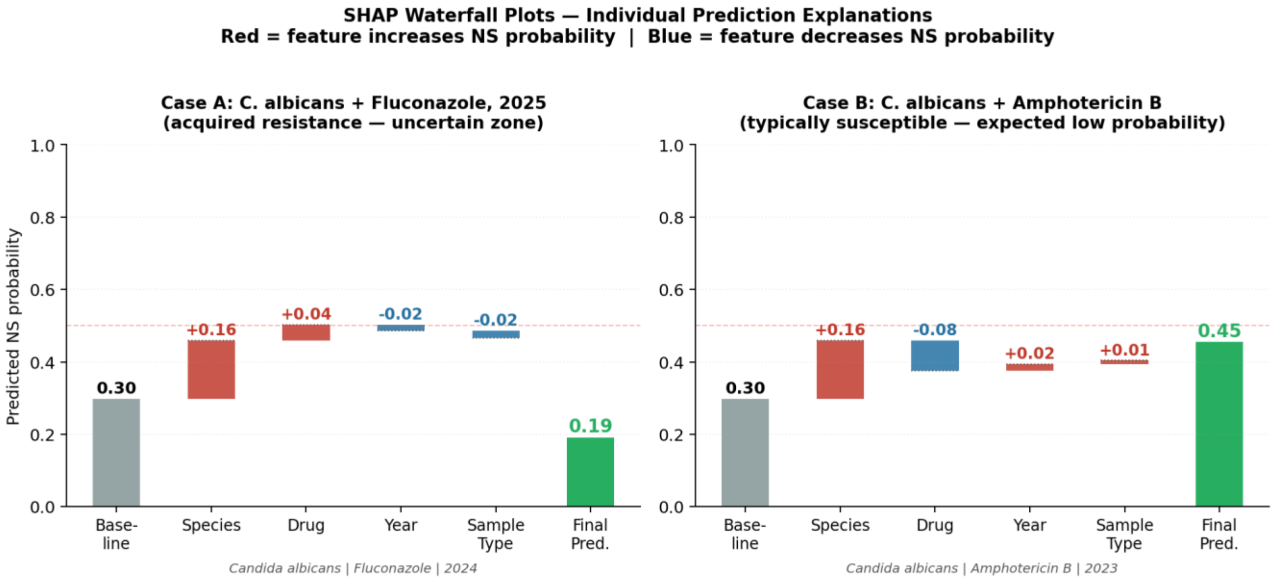

*Supplementary Figure S10**.** SHAP Waterfall Plots. Individual Prediction Explanations. Feature-level contribution to predicted non-susceptibility probability for two representative isolate–drug observations: Case A (C. albicans + Fluconazole, 2024) and Case B (C. albicans + Amphotericin B, 2023). Red bars indicate features that increase predicted probability; blue bars indicate features that decrease it relative to the model baseline (0.298)*
